# Guanidine *N*-methylation by BlsL Is Dependent on Acylation of Beta-amine Arginine in the Biosynthesis of Blasticidin S

**DOI:** 10.3389/fmicb.2017.01565

**Published:** 2017-08-22

**Authors:** Xiankun Wang, Aiqin Du, Guiyang Yu, Zixin Deng, Xinyi He

**Affiliations:** State Key Laboratory of Microbial Metabolism, School of Life Sciences and Biotechnology, Shanghai Jiao Tong University Shanghai, China

**Keywords:** arginine *N*-methyltransferase, blasticidin S biosynthesis, nucleoside resistance, leucyldemethylblasticidin S, substrate preference

## Abstract

The peptidyl nucleoside blasticidin S (BS) produced by *Streptomyces griseochromogenes* was the first non-mercurial fungicide used to prevent rice blast and increasingly used as a selection reagent in transgenic study. Acylation by addition of a leucine residue at the beta amine group of arginine side chain of demethylblasticidin S (DBS) has been proposed as a novel self-resistance to the cytotoxic biosynthetic intermediate. But the resultant product leucyldemethylblasticidin S (LDBS) has not been isolated as a metabolite, and LDBS synthetase activity remained to be demonstrated in *S. griseochromogenes*. In this study, we isolated LDBS in a BS heterologous producer *S. lividans* WJ2 upon the deletion of *blsL*, which encodes a S-Adenosyl methionine-dependent methyltransferase. Purified BlsL efficiently methylated LDBS at the delta N of beta-arginine to generate the ultimate intermediate LBS, but nearly didn’t methylate DBS to final product BS. Above experiments demonstrated that LDBS is indeed an intermediate in BS biosynthetic pathway, and acylation of beta-amino group of arginine side chain is prerequisite for efficient guanidine *N*-methylation in addition to being a self-resistance mechanism.

## Introduction

Blasticidin S (BS) was first isolated from the broth of *Streptomyces griseochromogenes* as an effective non-mercurial fungicide (Takeuchi et al., 1958). BS displayed wide-range antimicrobial spectrum, it exerts its biological activities by inhibiting protein translation through a unique mechanism by which it traps the deformed P-site tRNA and strongly suppresses peptidyl-tRNA hydrolysis by release factors and peptide bond formation (Svidritskiy et al., 2013). As it’s friendly to environment and not harmful to fish, blasticidin S was soon used on a large scale by foliar application in Asia to control rice blast caused by *Pyricularia oryzae* (Misato et al., 1958). Now it has been increasingly used as the selection reagent in mammalian transgenic studies (Kamakura et al., 1990; Freitas et al., 2002). Moreover, BS strongly inhibits the production of aflatoxin by fungus *Aspergillus flavus* that is toxic and highly carcinogenic (Yoshinari et al., 2013).

Blasticidin S is consisted of a *N*-methyl-guanidine tail attached to a pyranose ring and bonded to a cytosine (Ōtake et al., 1966), its core moiety cytosylglucuronic acid (CGA) is shared by mildiomycin, gougerotin, arginomycin (Fox et al., 1968; Harada et al., 1978; Argoudelis et al., 1987). Unfortunately, studies on BS biosynthesis have proceeded slowly since the cloning of BS biosynthetic gene cluster as gene deletion in BS native producer *S. griseochromogenes* was never successful. In order to bypass the restriction barrier, we engrafted BS biosynthetic gene cluster onto the chromosome of *S. lividans* and generated a BS heterologous producer *S. lividans* WJ2 (Li et al., 2013), which here was used for *in vivo* study.

The putative BS biosynthetic pathway was proposed based on feeding experiments (Seto et al., 1968) and determination of metabolites (Prabhakaran et al., 1988; Guo and Gould, 1991; Gould and Zhang, 1995). However, several steps and their catalyzed enzymes remain to be established. A most intriguing question regarding BS biosynthesis is BS maturation. Demethylblasticidin S (DBS) had been isolated as a minor metabolite in the broth of *S. griseochromogenes*, and was proposed as the penultimate metabolite for *N*-methylation to give the final BS. But in the presence of S-Adenosyl methionine (SAM), the CFE of *S. griseochromogenes* could only methylate DBS at a ratio of as low as 0.85%. In contrast, a derivative named AcDBS, with the beta amino group of guanidine acetylated, could be methylated to form AcBS at a ratio of 21% (Zhang et al., 2000). However, AcBS cannot be hydrolyzed by the CFE of *S. griseochromogenes* to form mature BS, therefore both DBS and AcDBS were excluded as the direct precursors for *N*-guanidine methylation. On the other hand, leucylblasticidin S (LBS) was sometimes isolated as an intermediate in the broth of *S. griseochromogenes* when the pH of the culture medium was kept below 4 (Seto et al., 1968), it was later isolated from *Streptomyces* sp. SCC 1785 (Cooper et al., 1988) and *S. griseochromogenes* under normal laboratory conditions (Zhang et al., 2000). Discovery of LBS as the metabolite implied that the real intermediate for *N*-methylation might be LDBS rather than DBS. Interestingly, chemically synthesized LDBS displayed 20-fold less inhibition activity against *Bacillus circulans* than DBS. Therefore, leucylation of DBS into LDBS was postulated to be a novel self-resistance mechanism to protect the host from the toxic biosynthetic intermediate. However, the intermediacy of LDBS in BS biosynthesis remains elusive because LDBS is rarely detected in the native producer *S. griseochromogenes*, and the attempts to demonstrate LDBS synthetase activity were not successful (Zhang et al., 2000).

Here, we isolated LDBS as a major metabolite upon the deletion of the SAM-dependent guanidine methyltransferase gene *blsL* in a BS heterologous producer *S. lividans* WJ2. Moreover, BlsL can efficiently methylate LDBS into LBS, similar to methylation of the acetyl-DBS, but nearly cannot methylate DBS, demonstrating acylation of beta-amine is prerequisite for efficient methylation of guanidine *N*-methylation in BS biosynthesis beyond being as a self-resistance mechanism.

## Materials and Methods

Compound Blasticidin S was purchased from YEASEN Biotech Co., Ltd. (Shanghai, China). DBS, LDBS, and LBS were synthesized by Professor Mark Zabriskie at Oregon State University (Zhang et al., 2000). SAM, L-arabinose, apramycin, spectinomycin, kanamycin and trimethoprim (TMP) were purchased from Sangon Biotech Co., Ltd. (Shanghai, China). *S. lividans* WJ2 contains the BS biosynthetic gene cluster and capable of producing blasticidin S (Li et al., 2013), *S. lividans* WXK1 is derived from WJ2 by deletion of *blsL* gene, *S. lividans* WXK2 is derived from WXK1 complemented with *blsL* in an integrative plasmid pIB139. All *Streptomyces* strains (Supplementary Table S1) were cultivated at 30°C on Soy Flour-Mannitol (SFM) agar plates for sporulation, mycelia were grown in 10.3% TSBY (tryptic soy broth supplemented with 10.3% sucrose (wt/vol) and 1% yeast extract (wt/vol) medium. *E. coli* strain DH10B was used for construction cloning vectors, BW25113/pIJ790 was used for in-frame deletion of *blsL* gene by PCR targeting, ET12567/pUZ8002 was used for conjugal transfer with *Streptomyces. E. coli* BL21(DE3) (Novagen) was used as a host for protein expression. *E. coli* strains (Supplementary Table S1) were cultivated at 37°C in LB/LA medium containing corresponding antibiotic. Plasmid pJTU1780 contains 35 kb fragment of BS biosynthetic cluster and pET28a (Novagen) is for protein overexpression.

### General Molecular Biology Methods

Plasmid DNA were purified by plasmid mini kit (Omega), isolation of genomic DNA and other manipulations of *Streptomyces* were performed using the method described by Keiser et al. (2000). Restriction enzymes NdeI, EcoRI and HindIII and DNA polymerase were purchased from New England Biolabs (Beijing) Ltd. Primers (Supplementary Table S1) were synthesized by JIE LI BIOLOGY (Shanghai, China), PCR products were purified from agarose gels (0.8%) using DNA Gel Extraction Kit (Omega). T4 ligase kit for construction of recombinant plasmid was purchased from TaKaRa (China). The PCR-targeting method for in-frame deletion of *blsL* was done according to Bertolt Gust (Gust et al., 2002).

### HPLC and LC-MS Analysis

The acquisition of the fermentation products of *Streptomyces lividans* WJ2 and its derived strains were employed using secondary fermentation according to Li et al. (2013). After 2–6 days of fermentation, 25 ml fermentation broth was transferred to a 50 ml centrifuge tubes, 5000 r/min for 10 min, the supernatant was collected and adjusted to pH 5.0 using oxalic acid and then lay aside at room temperature for 30 min before centrifugation (10000 r/min, 30 min) again. The Supelclean LC-SCX solid-phase extraction (SPE) columns (bed weight, 500 mg; volume, 3 ml) (Supelco) was used for purification of BS and other intermediates. The column was activated by washing with 3 ml methanol and 5 ml sterile water, the supernatants were injected to penetrate the column and followed by injection of 5 ml sterile water to remove unwanted inclusions, then 5 ml 0.5% ammonia solution were flowed through the column to further remove impurities. Finally, 3 ml 5% ammonia solution were used to elute BS-related products. HPLC analysis was performed on an Agilent series 1260 using the innoval C18 column (4.6 mm × 250 mm, Agela Technologies). Two buffer were used, buffer A1: 0.1% (vol/vol) trifluoroacetic acid in water was aqueous phase, buffer B was organic phase (methanol). The samples were eluted at a flow rate of 0.3 ml/min and the concentration of buffer B rose from 5 to 40% in 40 min. The substances were detected at UV275. To confirm the molecular weight of each peak, a LC-MS analysis was done using an Agilent 1100 LC/MSD (mass-selective detector) with electrospray ionization (ESI) in the positive mode.

### Overexpression and Purification of BlsL

High-fidelity DNA polymerase Hieff TM Pfu (YEASEN BiotechCo., Ltd., Shanghai, China) was used to amplify the *blsL* gene sequence from plasmid pJTU1780 with the Forward primer: GGAATTCCATATGGAGCAGAGCACCGGCGCGAT and Reverse primer: CGGAATTCTCAGTCCTTGTAGAAACACT (NdeI and EcoRI sites are underlined) by PCR. The PCR product were purified and digested by NdeI and EcoRI and ligated with pET28a vector which was also cut by NdeI and EcoRI, the ligation mixture was transformed into *E. coli* DH10B. After confirmation of the right clone by sequencing, recombinant plasmid pET28a-*blsL* was transformed into *E. coli* BL21(DE3) and grown overnight at 37°C in 10 ml LB medium supplied kanamycin (final concentration 50 μg/ml). 10 ml of cell culture were inoculated in 1 L LB medium in a 3 L flask and cultured at 37°C until OD600 approaching 0.6 and then 250 μl isopropyl-D-thiogalactopyranoside (IPTG, 800 mM) was added to a final concentration of 0.2 mM, the culture medium was then transferred to 16°C and grown for 16 h. Cells were harvested by centrifugation at 5000 r/min for 10 min and suspended in 50 ml of lysis buffer (50 mM Tris–HCl pH 7.8, 0.5 M NaCl and 20 mM imidazole) and lysed by high power press cell disrupter (UH-240/UH-480/UH-960, Union-Biotech, Co., Ltd.) at 800 bar for 5 min. Then centrifugation at 10000 r/min for 1 h and the supernatant were applied to a column filled with 3 ml Ni-NTA agarose (GE Healthcare), washing the column with 60 ml washing buffer (50 mM Tris–HCl pH 7.8, 0.5 M NaCl and 40 mM imidazole) and eluted the His-tagged protein BlsL with 15 ml elution buffer (50 mM Tris–HCl pH 7.8, 0.5 M NaCl and 300 mM imidazole). The eluted BlsL was desalted using PD10 desalting column (GE Healthcare) and stored in storing buffer (25 mM Tris–HCl buffer pH 7.8, 100 mM NaCl and 10% glycerol). The purity of BlsL was detected by SDS–PAGE and the concentration of BlsL was determined by UV absorption at A280 using NanoDrop (Thermo).

### *In Vitro* Assay of the Purified BlsL

The *in vitro* assay of BlsL was carried out in reaction buffer (25 mM Tris–HCl buffer, pH 7.8 and 100 mM NaCl) at 37°C for 1 h or overnight, the reaction volume was 100 μl with 10 μM BlsL, 1 mM DBS or 1 mM LDBS and 2 mM SAM. The reactions were quenched by adding equal volume of chloroform and shocked for 1 min to denature the BlsL and the supernatant was collected by centrifugation (12000 r/min, 10 min). The measurement of the reaction products was performed by HPLC and LC-MS using TC-C18 (4.6 mm × 250 mm, Agilent). Aqueous phase was buffer A2 (20 mM aqueous phase, pH 5.5), organic phase was still buffer B, the sample were eluted at a flow rate of 0.3 ml/min with buffer B rising from 10 to 50% in 30 min.

## Results

### Analysis of BlsL, a SAM-Coordinated Methyltransferase in Blasticidin S Biosynthesis

**FIGURE 1:**
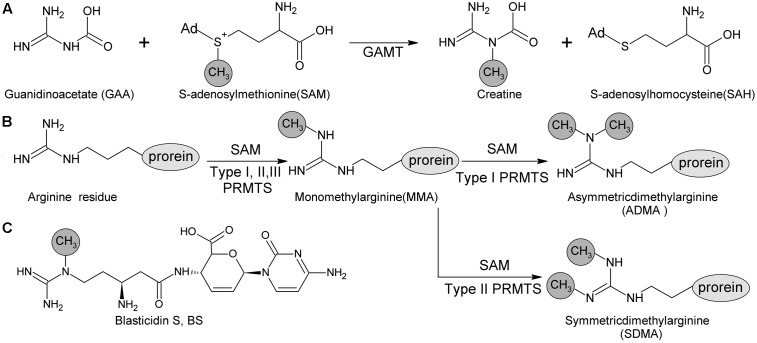
Methyl -transfer reaction catalyzed by GMAT and PRMTs. **(A)** Guanidino acetate methyltransferase (GAMT) catalyzes the guanidinoacetate (GAA) and S-adenosyl methionine (SAM) into creatine and S-adenosylhomocysteine (SAH). **(B)** Protein arginine methyltransferase (PRMT) family containing three types (Types I, II and III) according to the type of methylation they catalyze. All of these types produce ω-NG–monomethylarginine (MMA); additionally, Type I PRMTs are responsible for producing ω-NG-NG–asymmetric dimethylarginine (ADMA) and Type II PRMTs are for producing ω-NG-N′G-symmetric dimethylarginine (SDMA). **(C)** Methyl group at delta N of beta-amine arginine of blasticidin S is catalyzed by BlsL.

*blsL* is the sole methyltransferase gene in BS biosynthetic gene cluster, and thus proposed to be in charge of Guanidine-*N*-methylation. Sequence analysis of BlsL revealed that low similarity to guanidine acetate methyltransferase (GAMT, 33% identity, 85% similarity) and protein arginine methyltransferase (PRMT, 35% identity, 74% similarity). GAMT catalyzes guanidinoacetate (GAA) and SAM into creatine and S-adenosylhomocysteine (SAH). GAMT deficiency causes mental retardation, speech delay, and epilepsy (Velichkova and Himo, 2006). PRMT both catalyzes the formation of monomethylarginine (MMA) and symmetrical dimethylarginine (SDMA) from arginine residues of versatile proteins, and is involved in regulation of many cellular processes (Poulard et al., 2016). Both GAMT and PRMT catalyze *N*-methylation of guanidine group, but differs in the selection of target N atoms (**Figure 1**).

Sequence comparison revealed that BlsL contains a SAM-coordination motif E/DXGXGXGX that is conserve in above two protein families (Supplementary Figure S1) (Martin and McMillan, 2002). Corresponding to this, free SAM can be released from boiled BlsL that was heterologously expressed and purified in *E. coli*. Association of SAM with BlsL was confirmed by HPLC as well as MS/MS fragmentation (Supplementary Figure S2).

### LDBS Is Isolated As an Intermediate upon Deletion of *blsL* in a Blasticidin S Heterologous Producer

**FIGURE 2:**
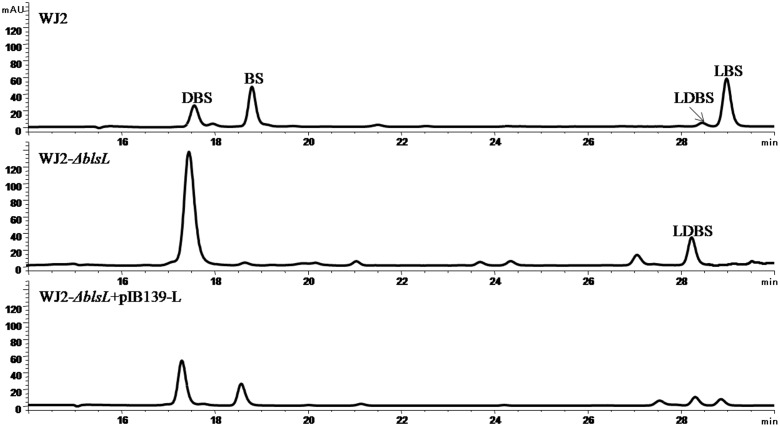
HPLC analysis of fermentation products of BS heterologous producer strain *S. lividans* WJ2 and two mutant strains WJ2 (Δ*blsL*) and WJ2 (Δ*blsL*)::*blsL*. The column used for analysis of metabolites here is Innoval C18 column (4.6 mm × 250 mm, Agela Technologies), and the elution conditions please refer to the Section “Materials and Methods.”

As genetic manipulation in BS native producer *S. griseochromogenes* has been not successful. We studied BS biosynthesis in a heterologous producer *S. lividans* WJ2 (Li et al., 2013). In order to know the role of BlsL in the biosynthesis of BS, *blsL* was in-frame deleted in WJ2 to generate mutant WXK1. Compared with WJ2, two peaks in HPLC assay corresponding to LBS and BS disappeared in the fermentation profile of mutant WXK1 whereas the demethylated intermediates DBS and LDBS were retained and yields of them were significantly enhanced (**Figure 2**). Complementation of WXK1 with *blsL in trans* under constitutive promoter *ermE^∗^* on integrative plasmid pIB139 can restore the mutant production of LBS and BS. These demonstrating that BlsL is the only enzyme in WJ2 governing delta-N methylation of guanidine side chain, the detection of LDBS, especially in mutant WXK1 implied that LDBS is a true intermediate for BS biosynthesis.

### BlsL Effectively Catalyzes Guanidine *N*-methylation of LDBS

**FIGURE 3:**
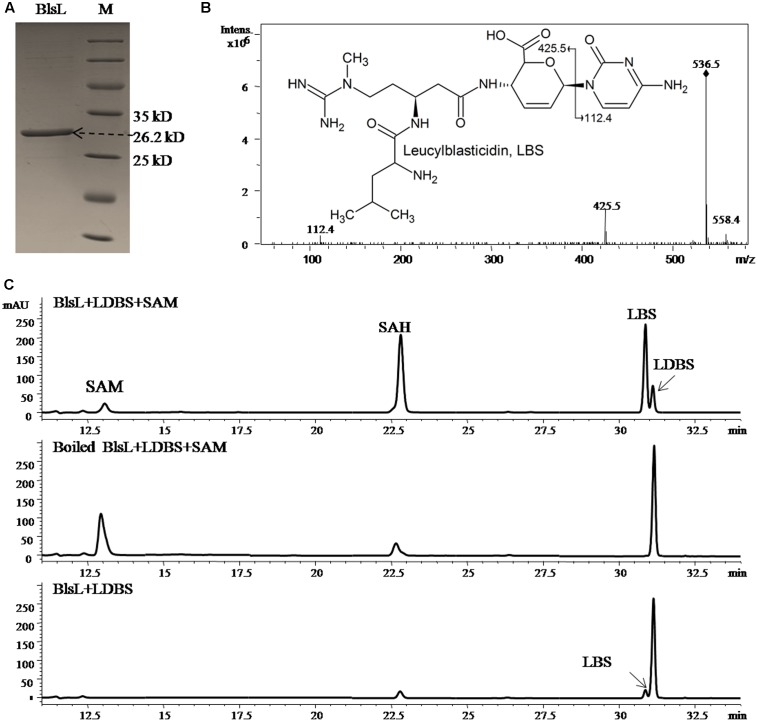
BlsL efficiently catalyses the formation of LBS with LDBS as the substrate. **(A)** SDS–PAGE analysis of purified BlsL; **(B)** MS/MS fragments of LBS generated by BlsL-catalyzed methylation with LDBS and SAM, **(C)** HPLC analysis of the reaction products of BlsL with LDBS and SAM. The measurement of the reaction products were performed by HPLC and LC-MS using TC-C18 (4.6 mm × 250 mm, Agilent) and the elution conditions please refer to the Section “Materials and Methods.”

The co-existence of DBS and LDBS in fermentation broth of both WJ2 and WXK1 made it hard to know which one is the true substrate for BlsL. In order to address this question, we measured the methyltransferase activity of purified BlsL on chemically synthesized LDBS and DBS (Gifts from Prof. Mark Zabriskie) (Zhang et al., 2000). Compared to the boiled BlsL, purified BlsL (**Figure 3A**) can convert 80% of LDBS into LBS in 1-h incubation; concomitantly, 90% of SAM were consumed and turned into SAH (**Figure 3B**). We also noticed that the control reaction without SAM also gave 5% conversion of LDBS into LBS, whose structure was confirmed by secondary MS fragmentation (**Figure 3C**), consistently supporting that purified BlsL contains SAM and catalyzes the *N*-methylation of LDBS.

### BlsL Nearly Doesn’t Methylate DBS

**FIGURE 4:**
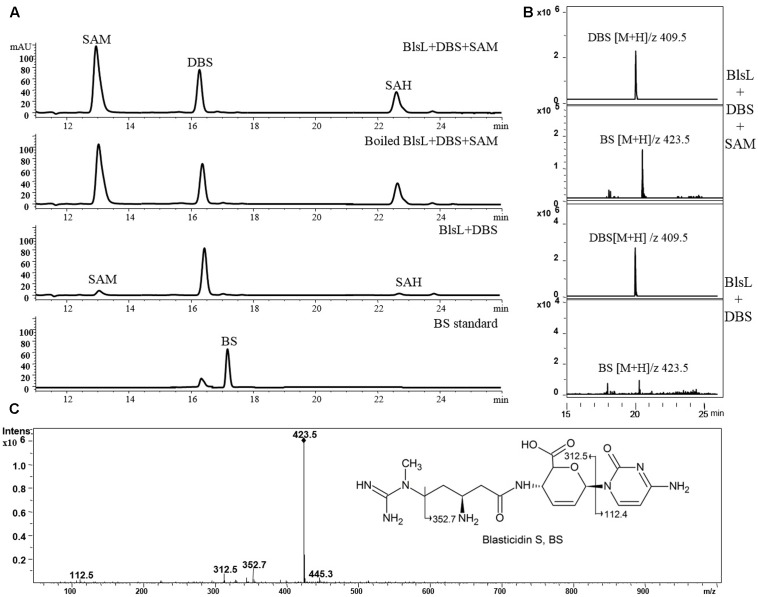
BlsL nearly cannot catalyze the formation of BS with DBS as the substrate. **(A)** HPLC analysis of the reaction products of BlsL with DBS and SAM; **(B)** Increased intensity of ion for BS detected in the reaction of BlsL, DBS and SAM compared with boiled control protein; **(C)** MS/MS fragments of BS generated by BlsL-catalyzed methylation with DBS and SAM.

In sharp contrast, two substrates DBS and SAM both kept intact after 1-h reaction with BlsL, and there is no visible peak in HPLC corresponding to BS, for which DBS was previously proposed to be the direct substrate (**Figure 4A**). These results demonstrated that DBS cannot be catalyzed by BlsL. However, MS spectrum analysis detected the ion at M/Z 423 that corresponds to the mass for BS (**Figure 4B**), secondary MS fragmentation pattern further supported the formation of BS (**Figure 4C**), consistent with the result that only 0.85% of DBS can be converted to BS by the cell of *S. griseochromogenes* (Zhang et al., 2000).

**FIGURE 5:**
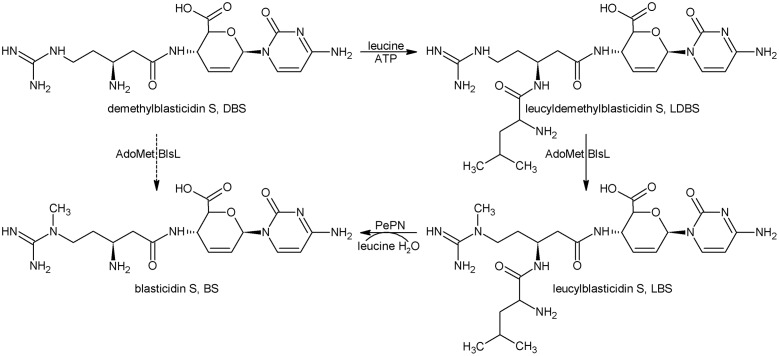
The last four biosynthetic steps for BS. DBS, Demethylblasticidin S; LDBS, leucyldemethylblasticidin; LBS, leucylblasticidin S; BS, blasticidin S.

Taken together, the last four biosynthetic steps are summarized as in **Figure 5**. Namely, DBS can nearly not be directly converted into BS whereas LDBS is the true substrate for BlsL, and thus demonstrating that LDBS is the real intermediate for methylation in BS biosynthesis.

## Discussion

In the study of BS biosynthesis, Seto and Yonehara (1977) isolated intermediate DBS and a minor metabolite LBS in *S. griseochromogenes*. Both compounds require a single transformation for conversion to BS (**Figure 5**). DBS was assayed as the precursor for BS by using the Cell-Free-Extract of *S. griseochromogenes*, but the SAM and DBS both were not consumed. So DBS is not the direct precursor for the guandino methylase. In the light of biosynthetic study of another nucleoside puromycin, the 2″-amino group of *O*-methyl-L-tyrosine moiety was acetylated in the presence of acetyl coenzyme A, and was followed by methylation (Pérez-González et al., 1983; Sugiyama et al., 1985).

The compound acetyl-DBS (AcDBS) with the beta-amino group of arginine side chain by acetylated was assayed and found to be efficiently converted to AcBS, implied that acetylation of beta-arginine is essential to the guanidine *N*-methylation. However, AcDBS couldn’t be hydrolyzed into mature BS, and thus is not the biosynthetic intermediate for BS.

The switching relationship between two metabolite DBS and LBS requires leucylation of DBS to form LDBS followed by methylation of guanidine group (**Figure 5**). Chemically synthesized LDBS showed decreased cytotoxicity by 20-folds compared to DBS, and thus leucylation of DBS was regarded as a novel self-resistance because of inactivation of an antibiotic with an amino acid (Zhang et al., 2000). This proposal was challenged by the failures both in isolations of intermediate LDBS and confirmation of LDBS synthetase activity in *S. griseochromogenes*. In view of present situation of BS biosynthesis, failure in isolation of LDBS and only accumulation of very minor of LBS in the broth of *S. griseochromogenes* are attributed to efficient excision of leucine group from above two metabolites by standalone PepN and its homologs (Yu et al., 2015). Different from in the BS native producer, LBS is often presented as the dominant metabolite in a heterologous producer *S. lividans* WJ2, particularly when the pH of broth was kept below 6, this case usually occurred when the medium is contaminated in later phase of fermentation by acid-producing bacteria, such as *E. coli*. Also, LDBS could also be detected in WJ2, its production was apparently enhanced when the methyltransferase gene *blsL* was deleted (**Figure 2**). Furthermore, purified BlsL *in vitro* can effectively catalyze LDBS into LBS in the presence of SAM, but not methylate DBS, thus demonstrating the intermediacy of LDBS in BS biosynthesis.

Acylation of DBS at the beta-amine including AcDBS as well as LDBS significantly increased the methylation efficiency by BlsL *S. griseochromogenes*, implying that acylation of beta-amine might change the distribution of the charge or provide better fit each other between BlsL and the substrate either in recognition or catalysis. This work provided a solid base for demonstration of last four biosynthesis steps in BS biosynthesis.

## Author Contributions

XW and AD performed experiments. GY provided compounds and strains. XW, XH, and ZD analyzed the data. XH wrote the manuscript. All authors reviewed, revised, commented on and approved the final version of the manuscript.

## Conflict of Interest Statement

The authors declare that the research was conducted in the absence of any commercial or financial relationships that could be construed as a potential conflict of interest.
