# Supplementary material for: Guanidine N-methylation by BlsL Is Dependent on Acylation of Beta-amine Arginine in the Biosynthesis of Blasticidin S
Source: Front Microbiol. 2017 Aug 22;8:1565. doi: 10.3389/fmicb.2017.01565 (PMC5572114; doi:10.3389/fmicb.2017.01565)
Supplement: Supplementary file 1 [file Table_1.docx]

**Table S1 Strains, plasmids and primers used in this study.**

| **Strains** | **Relevant properties** | **Source** |
| --- | --- | --- |
| *Streptomyces lividans*WJ2 | Blasticidin S heterologous expression  strain | ^[^[^1^](#_ENREF_1)^]^ |
| *Streptomyces lividans*WXK1 | *blsL*in-frame deletion mutant | This study |
| *Streptomyces lividans*WXK2 | WXK1 with complemented with pIB139-*blsL* | This study |
| *Streptomyces griseochromogenes* | Native blasticidin S producer | CGMCG |
| *Escherichia coli* DH10B | F^-^ *rec*A*lac*Z△M15 | GIBCO BRL |
| *Escherichia coli* ET12567  /pUZ8002 | *rec*F, *dam, dcm, hsdS,* Cml^r^*,* Str^r^, Tet^r^, Km^r^ | ^[2]^ |
| *Escherichia coli* BL21 (DE3) | F^-^ ompTrB^-^mB^-^ (λDE3) | Novagen |
| *Escherichia coli* BW25113/pIJ790 | RepA101(ts),*araBp-gam-be-exo*,  AraC, RepA101(ts) Cml^r^ | ^[2]^ |
| **Plasmids** |  |  |
| pET28a | Expression vector with 6XHis-tag. | Novagen |
| pIJ778 | *aadA* resistance cassette | ^[3]^ |
| pIB139 | *attP, Int, oriT, PermE*, aac(3)IV* | ^[4]^ |
| pWXK1 | pIB139 overexpressing *blsL* | This study |
| **Primers** (from 5′ to 3′) | | |
| Tar-*blsL*-F | GCCGTCAGCCTCCTTCCCGTCCCGAGGAGGACAGTAATGATTCCGGGGATCCGTCGACC | |
| Tar-*blsL*-R | CTCCGACGCACTGCTGATGACGTTCACGAGGTTCCCTCATGTAGGCTGGAGCTGCTTC | |

**REFERENCE**

[1] Li, L., Wu, J., Deng, Z., Zabriskie, T. M. & He, X. *Streptomyces lividans* blasticidin S deaminase and its application in engineering a blasticidin S-producing strain for ease of genetic manipulation. Applied and environmental microbiology79, 2349-2357 (2013).

[2] Kieser, T., M. J. Bibb, K. F. Chater, M. J. Butter, and D. A. Hopwood.Practical *Streptomyces* genetics. The John Innes Foundation,United Kingdom, Norwich (2000).

[3] Gust, B., Challis, G. L., Fowler, K., Kieser, T. & Chater, K. F. PCR-targeted *Streptomyces* gene replacement identifies a protein domain needed for biosynthesis of the sesquiterpene soil odor geosmin. Proceedings of the National Academy of Sciences of the United States of America100, 1541-1546, doi:10.1073/pnas.0337542100 (2003).

[4] Wilkinson, C. J. et al. Increasing the efficiency of heterologous promoters in actinomycetes. Journal of molecular microbiology and biotechnology4, 417-426 (2002).

**Figure captions**

Figure S1 Multiple alignments of BlsL with its homologs that are annotated as the S-Adenosyl methionine(SAM)-dependent methyltransferases. A conserved SAM-coordination site EXGXGXG was identified and highlighted in red rectangle.

Figure S2 Confirmation of the presence of SAM in purified BlsL. a, UV detection of the absorption peak that corresponds to that of SAM standard in the boiled BlsL. b, MS fragmentation pattern of molecule for the absorption peak is same as that of SAM.
