# Supplementary figures and images for: Guanidine N-methylation by BlsL Is Dependent on Acylation of Beta-amine Arginine in the Biosynthesis of Blasticidin S
Source: Front Microbiol. 2017 Aug 22;8:1565. doi: 10.3389/fmicb.2017.01565 (PMC5572114; doi:10.3389/fmicb.2017.01565)

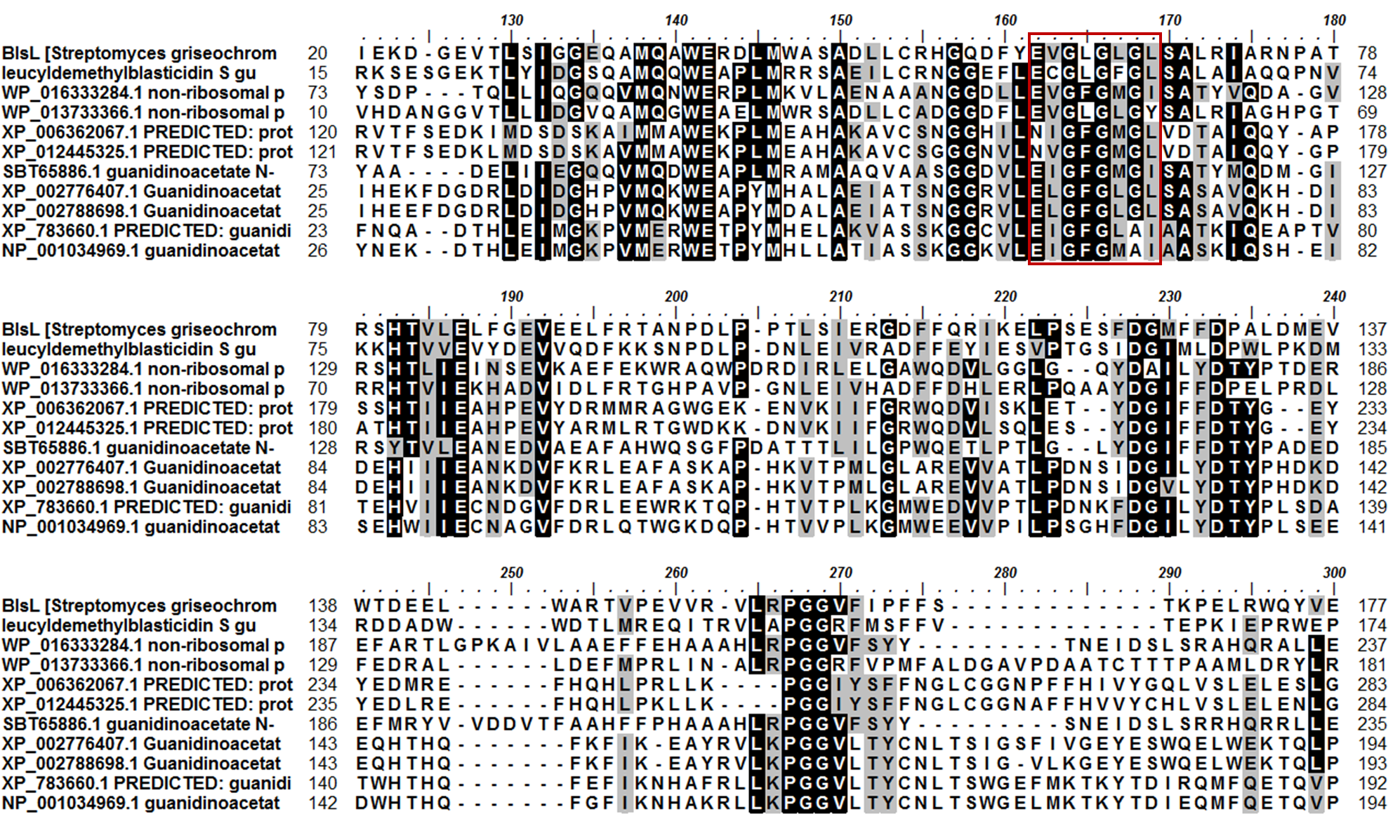

Supplement: Supplementary file 2 [file Image_1.TIF]

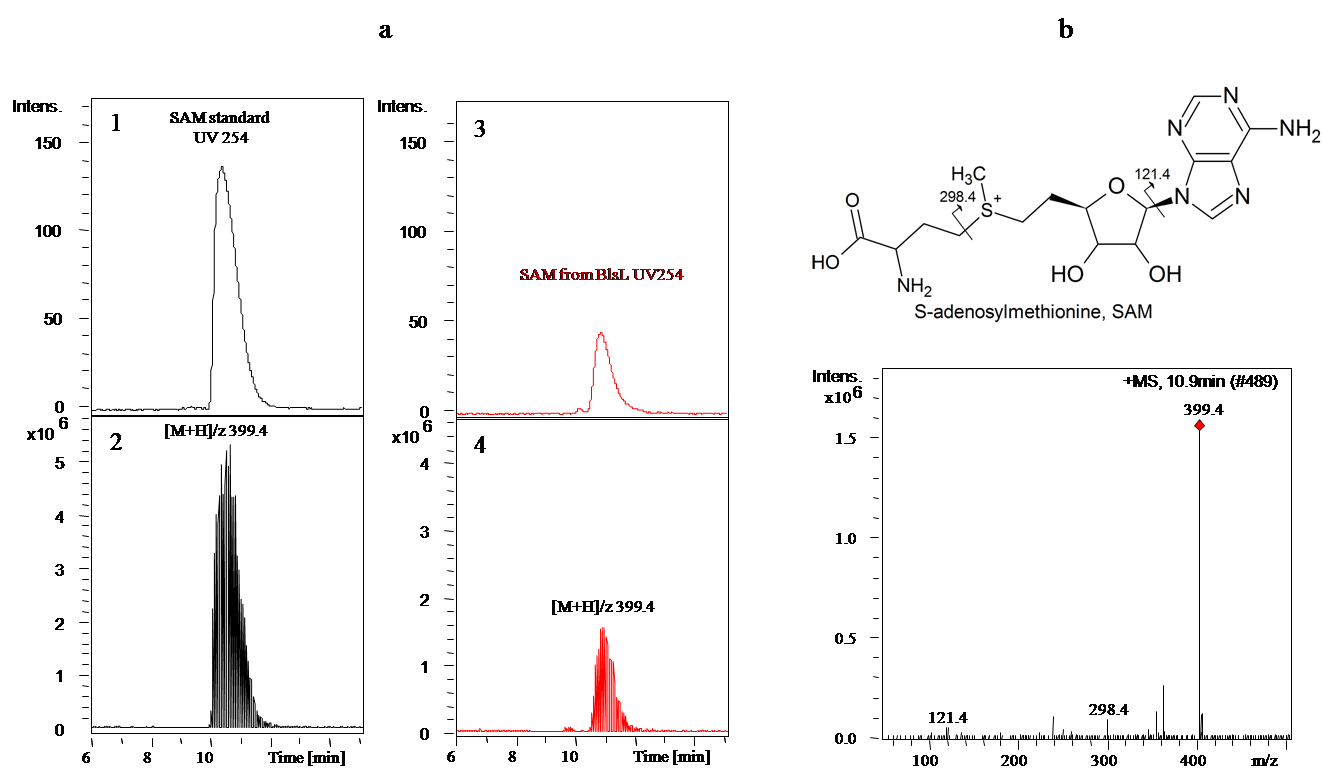

Supplement: Supplementary file 3 [file Image_2.TIF]
